# Stratifying nonfunctional pituitary adenomas into two groups distinguished by macrophage subtypes

**DOI:** 10.18632/oncotarget.26775

**Published:** 2019-03-15

**Authors:** Garima Yagnik, Martin J. Rutowski, Sumedh S. Shah, Manish K. Aghi

**Affiliations:** ^1^ Department of Neurosurgery, University of California San Francisco (UCSF), San Francisco, CA, USA

**Keywords:** macrophages, pituitary adenomas, M1, M2

## Abstract

Tumor-associated macrophages (TAMs) polarize to M1 and M2 subtypes exerting anti-tumoral and pro-tumoral effects, respectively. To date, little is known about TAMs, their subtypes, and their roles in non-functional pituitary adenomas (NFPAs). We performed flow cytometry on single cell suspensions from 16 NFPAs, revealing that CD11b^+^ myeloid cells comprise an average of 7.3% of cells in NFPAs (range = 0.5%–27.1%), with qPCR revealing most CD11b^+^ cells to be monocyte-derived TAMs rather than native microglia. The most CD11b-enriched NFPAs (10–27% CD11b^+^) were the most expansile (size>3.5 cm or MIB1>3%). Increasing CD11b^+^ fraction was associated with decreased M2 TAMs and increased M1 TAMs. All NFPAs with cavernous sinus invasion had M2/M1 gene expression ratios above one, while 80% of NFPAs without cavernous sinus invasion had M2/M1<1 (*P* = 0.02). Cultured M2 macrophages promoted greater invasion (*P* < 10^-5^) and proliferation (*P* = 0.03) of primary NFPA cultures than M1 macrophages in a manner inhibited by siRNA targeting S100A9 and EZH2, respectively. Primary NFPA cultures were of two types: some recruited more monocytes in an MCP-1-dependent manner and polarized these to M2 TAMs, while others recruited fewer monocytes and polarized them to M1 TAMS in a GM-CSF-dependent manner. These findings suggest that TAM recruitment and polarization into the pro-tumoral M2 subtype drives NFPA proliferation and invasion. Robust M2 TAM infiltrate may occur during an NFPA growth phase before self-regulating into a slower growth phase with fewer overall TAMs and M1 polarization. Analyses like these could generate immunomodulatory therapies for NFPAs.

## INTRODUCTION

Non-functional pituitary adenomas (NFPAs) are among the most common primary brain tumors [1]. While histologically benign, they radiographically and clinically range from slow growing, incidentally found tumors to more aggressive versions that can exert a devastating impact on a patient's quality of life through mass effect on neuroanatomical structures causing hypopituitarism [2], vision loss [3], and debilitating headaches [4].

While several studies have defined molecular alterations in NFPA cells [5–7], very few studies have investigated the immune microenvironment in NFPAs. One large study found that lymphocytic infiltrates are very rare in NFPAs and functional pituitary adenomas [8]. Another study identified elevated expression of programmed death ligand 1 (PD-L1) in pituitary adenomas, particularly in functional adenomas [9]. The ability of PD-L1 to allow cancers to escape T cell-mediated immune responses could explain the lack of lymphocytic infiltrates in pituitary adenomas.

In light of evidence pointing to an important role for macrophages in the initiation and progression of neoplasia [10], a study finding that larger adenomas have more tumor-associated macrophages (TAMs) [11] is intriguing. However, no study to date has accounted for the evolution of our understanding that the impact of TAMs on cancer progression is facilitated by the ability of tumors to hijack macrophages and convert them from default anti-tumoral phenotypes into pro-tumoral phenotypes capable of promoting tumor growth. In the case of TAMs, this alteration of function creates two subtypes of TAMs: M1 classically-activated macrophages with pro-inflammatory antitumoral function and M2 alternatively-activated macrophages which promote tumor growth and invasion and are associated with poor outcomes [12–14]. We therefore analyzed TAMs in NFPAs and delineated the role of M1 versus M2 TAMs in NFPA biology to determine if TAMs affected invasion or proliferation of NFPA cells.

## RESULTS

### Characterizing CD11b^+^ myeloid cells in NFPAs

**Figure 1:**
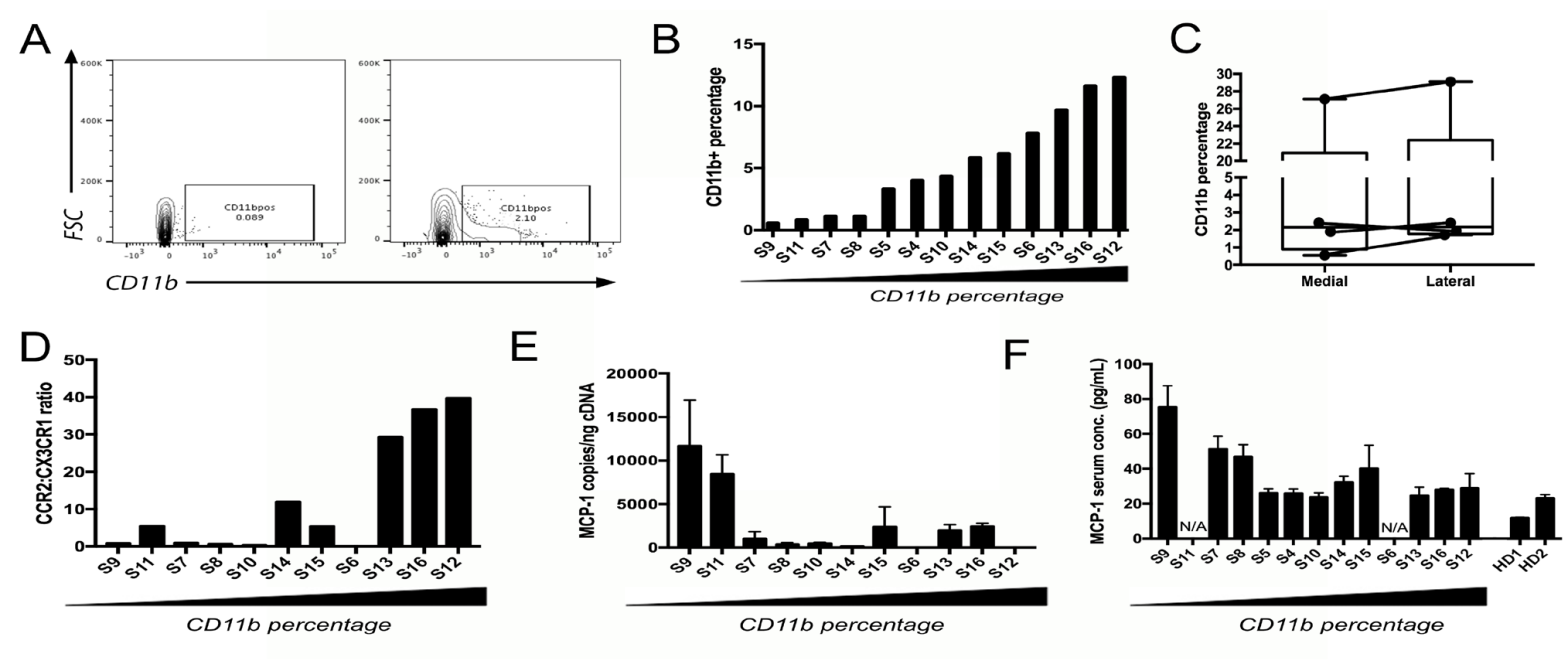
Characterizing CD11b^+^ myeloid cells in NFPAs (**A**) Representative flow cytometry scatter plots showing a single cell suspension of NFPA patient tumor both unstained (left) and stained (right) for CD11b cell surface marker. (**B**) Percentage of CD11b+ cells within flow cytometry sorted NFPA tumor samples, arranged from low to high percentage. (**C**) CD11b percentage from NFPA cases with site-directed biopsies obtained from the central portion of the adenoma (medial) and lateral edge of the adenoma abutting the medial cavernous sinus wall (lateral), showing no significant regional variation in macrophage percentages among biopsies (see Table 1). (**D**) qPCR of CD11b^+^ cells for TAM marker CCR2 and microglia marker CX3CR1 reveals that the ratio of CCR2 to CX3CR1 expression increases as the percentage of CD11b cells increases, consistent with these increased CD11b^+^ cells in NFPAs being TAMs. (**E**) Expression of MCP-1 in NFPA tumors dropped with increasing TAM levels (Pearson's correlation coefficient *r* = −0.482, *P* = 0.1). (**F**) Serum MCP-1 from the same NFPA patients whose samples were utilized for flow cytometry also dropped with increasing TAM levels (Pearson's correlation coefficient for: CD11b% vs. ELISA: *r* = −0.622, *P* = 0.04; PCR vs. ELISA: *r* = 0.764, *P* = 0.02). Serum from two healthy donors (HD) was run in parallel as controls. N/A = serum not available.

Patient tumor samples from 16 NFPAs obtained from the operating room were dissociated to single cell suspensions. These suspensions subsequently underwent flow cytometry for CD11b^+^ myeloid cells (Figure 1A), revealing that the percentage distribution of CD11b^+^ cells in NFPAs was skewed to the right with a mean (7.3%) greater than its median (5.1%) (range 0.5%–27.1%; Figure 1B), with 11 cases under 8% CD11b fraction and five cases exhibiting 10-27% CD11b fractions (Table 1). These five CD11b-rich NFPAs were the most expansile tumors (size > 3.5 cm or MIB1 > 3%; Table 1). While findings from flow cytometry did not correlate well with CD11b immunohistochemistry (Supplementary Figure 1), we focused on flow cytometry for subsequent analyses for two reasons. First, CD11b immunohistochemistry on paraffin-embedded sections has been shown to produce variable results based on processing and storage conditions [15, 16]. Second, immunohistochemistry has been argued to be inferior to flow cytometry for analysis of some cell types in tumors [17]. We also found that the percentage of cells in NFPAs that were CD11b^+^ did not vary regionally between the medial versus lateral aspects of the tumor (Figure 1C; Supplementary Figure 2), further validating the choice of this approach.

**Table 1:** Summary of macrophage profile from 20 nonfunctional pituitary adenomas

Sample	Gender	Age	Tumor diameter (cm)	Cavernous sinus invasion	MIB-1 (%)	% CD11b positive cells	Log (M2/M1 ratio)	% M1 (CD11b^+^ CD64^+^ CD206^-^)	% M2 (CD11b^+^ CD64^-^ CD206^+^)
S1	M	71	1.1	Yes	3%	2.7%	0.4	N/A	N/A
S2	F	70	2.5	Yes	10%	18.1%	2.2	N/A	N/A
S3	F	60	3.7	Yes	2%	28.1%	2.6	N/A	N/A
S4	M	63	2	No	1%	4.0%	N/A	5.7%	6.1%
S5	M	65	3.1	Yes	1%	3.3%	N/A	1.1%	1.8%
S6	F	31	1.5	Yes	1%	7.8%	7.3	1.2%	21.7%
S7	M	41	2.8	No	2.5%	1.1%	-6.4	2.3%	17.3%
S8	M	62	2.7	Yes	1%	1.1%	0.6	9.9%	0.5%
S9	F	75	2.3	No	1%	0.5%	-0.1	0.01%	64.3%
S10	M	24	3.0	No	3%	4.3%	-0.5	8.9%	0.7%
S11	F	30	0.8	No	1.5%	0.8%	-0.1	48.8%	10%
S12	M	75	1.6	Yes	3.5%	12.3%	3.0	3.9%	0.7%
S13	F	45	2.2	Yes	3.5%	9.7%	5.8	33.8%	4.5%
S14	M	58	0.8	Yes	3%	5.8%	0.2	18.6%	1.2%
S15	M	71	2.9	Yes	1.5%	6.1%	-0.1	0.5%	0.3%
S16	F	52	3.6	No	1%	11.6%	0.7	1.1%	0.1%
**AVERAGE/ TOTAL**	**7 F/9 M**	**56**	**2.3**	**10/16 (63%) with invasion**	**2.5%**	**7.3%**	**Not applicable**	**10.5%**	**9.9%**
*Site-directed biopsy (SDB) case*	*Gender*	*Age*	*Tumor Diameter (cm)*	*Cavernous Sinus Invasion*	*MIB-1 (%)*	*%CD11b positive cells*		*% M1 (CD11b+ CD64+ CD206-) and % M2 (CD11b+ CD64- CD206+)*	
						Medial	Lateral	Medial	Lateral
SDB1	F	60	3.7	Yes	2%	27.1%	29.1%	N/A	N/A
SDB2	F	31	1.2	Yes	8%	1.9%	2.4%	6.3% (M1); 2.4% (M2)	6.3% (M1); 1.5% (M2)
SDB3	M	59	3.1	No	4%	0.6%	1.7%	15.7% (M1); 0.4% (M2)	5.0% (M1); 0.2% (M2)
SDB4	M	46	2.0	No	3%	2.4%	2.0%	14.8% (M1); 1.4% (M2)	23.4% (M1); 1.9% (M2)
**AVERAGE/ TOTAL**	**2 F/2 M**	**49**	**2.5**	**2/4 (50%) with invasion**	**4.3%**	**8.0%**	**8.8%**	**12.3% (M1); 1.4% (M2)**	**11.6% (M1); 1.2% (M2)**

Shown are the gender, age, mean tumor diameter (in cm), cavernous sinus invasion, MIB-1 labeling index (%), percentage of cells in single cells suspensions made from NFPAs that were CD11b^+^ cells by flow cytometry, logarithm of the M2/M1 gene expression ratio assessed by qPCR of CD11b^+^ cells, percentage of CD11b^+^ cells that were M1 TAMs by flow cytometry, and percentage of CD11b^+^ cells that were M2 TAMs by flow cytometry from the 16 NFPAs in the 16 samples analyzed from a single pooled sample collected from throughout the tumor (S1-S16) and four cases analyzed by a pair of regional site-directed biopsies taken from the central portion of the adenoma and from the medial cavernous sinus wall (SDB1-SDB4). Note that one sample (S3) underwent SDBs which generated samples SDB1-2 which were analyzed individually and in a pooled manner to generate S3 results. Abbreviations used: N/A= not available.

### Origin of CD11b^+^ cells in NFPAs

We next established whether these CD11b^+^ cells in NFPAs were tumor-associated macrophages (TAMs) derived from either circulating marrow-derived monocytes recruited to NFPAs due to the lack of a blood-brain barrier in the pituitary or from microglia native to the CNS. We determined this by performing absolute qPCR for microglia marker CX3CR1 and myeloid macrophage marker CCR2 [5, 18, 19] on CD11b^+^ cells isolated from the NFPAs analyzed above. The ratio of CCR2 to CX3CR1 expression increased as the percentage of CD11b cells increased (Figure 1D), consistent with these increased CD11b^+^ cells in NFPAs being TAMs. Interestingly, expression of MCP-1 (monocyte chemoattractant protein; gene name *CCL2*), the most potent tumor-secreted monocyte-attracting chemokine [20], dropped in these NFPAs with increasing TAM levels (Figure 1E; Pearson's correlation coefficient for CD11b% vs. PCR: *r* = −0.482, *P* = 0.1), a finding that became significant when MCP-1 levels in in blood serum from these patients was quantified using ELISA (Figure 1F; Pearson's correlation coefficient for: CD11b% vs. ELISA: *r* = −0.622, *P* = 0.04; PCR vs. ELISA: *r* = 0.764, *P* = 0.02).

### Characterizing TAM subtypes in NFPAs

**Figure 2:**
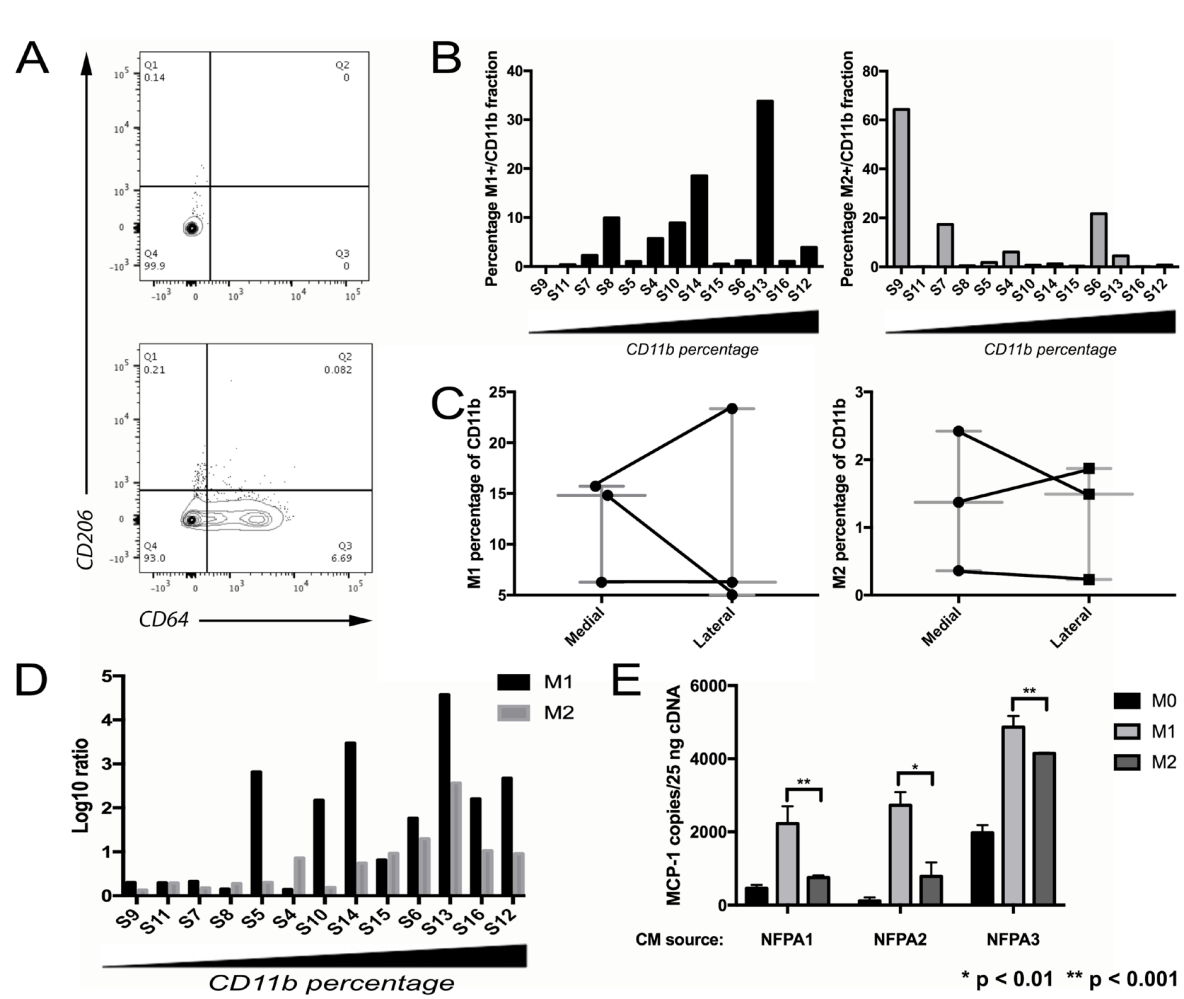
Characterizing TAM subtypes in NFPAs (**A**) Representative flow cytometry scatter plots showing CD11b^+^ fraction of an NFPA patient tumor cell suspension, either unstained (left) or stained (right) for M1 marker CD64 and M2 marker CD206. (**B**) NFPA tumor samples arranged from low to high percentage CD11b+ with percentage positive for M1 or M2 marker by flow cytometry reveals an increasing M1 percentage as the samples become more TAM enriched. (**C**) NFPA cases with site-directed biopsies were sorted using flow cytometry for polarized macrophages (Left: M1: CD11b+CD206-CD64+; Right: M2, CD11b+CD206+CD64-), which showed some regional variation in both M1 and M2 percentages, in both the medial and lateral regions of the tumor (see Table 1). (**D**) Results of qPCR performed on M1 and M2 sorted cells. These fractions from each sample were screened for the six previously described M1/M2 markers, followed by calculation of the log ratio of gene expression in markers from the group being screened vs. from the opposing group (**E**) CM from M2 macrophages reduced MCP-1 expression in cultured NFPA cells compared to conditioned media from M1 macrophages (Student's *t*-test, *P* < 0.01).

NFPAs were flow sorted for markers of M1 and M2 polarization within the CD11b+ population [21] (M1: CD11b^+^CD206^-^CD64^+^; M2: CD11b^+^CD206^+^CD64^-^; Table 1; Figure 2A). Increasing CD11b cell fraction was associated with an increased percentage of flow-sorted M1 TAMs and decreased percentage of flow-sorted M2 TAMs (Figure 2B). Because this percentage of M1 and M2 TAMs analyzed by flow cytometry showed some regional variation between the medial versus lateral aspects of NFPAs (Figure 2C) and because of literature supporting the complexity of M1 versus M2 phenotypes [22], we expanded our approach to include qPCR verification of the flow sorted M1 and M2 subpopulations. This was done utilizing previously described M1 (*NOS2, CXCL10, IL-1B*) and M2 (*ARG1, MMP9, TGFB1*) markers [23]. The qPCR approach verified that these markers correlated with the cell surface markers used to indicate polarization and confirmed the flow cytometry result that the relative levels of M1 markers outdistanced those of the M2 markers as CD11b+ percentage increased (Figure 2D). M2 TAMs also proved to be a source of the MCP-1 suppression we noted in the presence of increasing TAMs (Figure 1F) as we found that conditioned media (CM) from M2 macrophages reduced MCP-1 expression in cultured NFPA cells compared to CM from M1 macrophages (Figure 2E; *P* < 0.01).

### Effects of TAMs on NFPA proliferation

**Figure 3:**
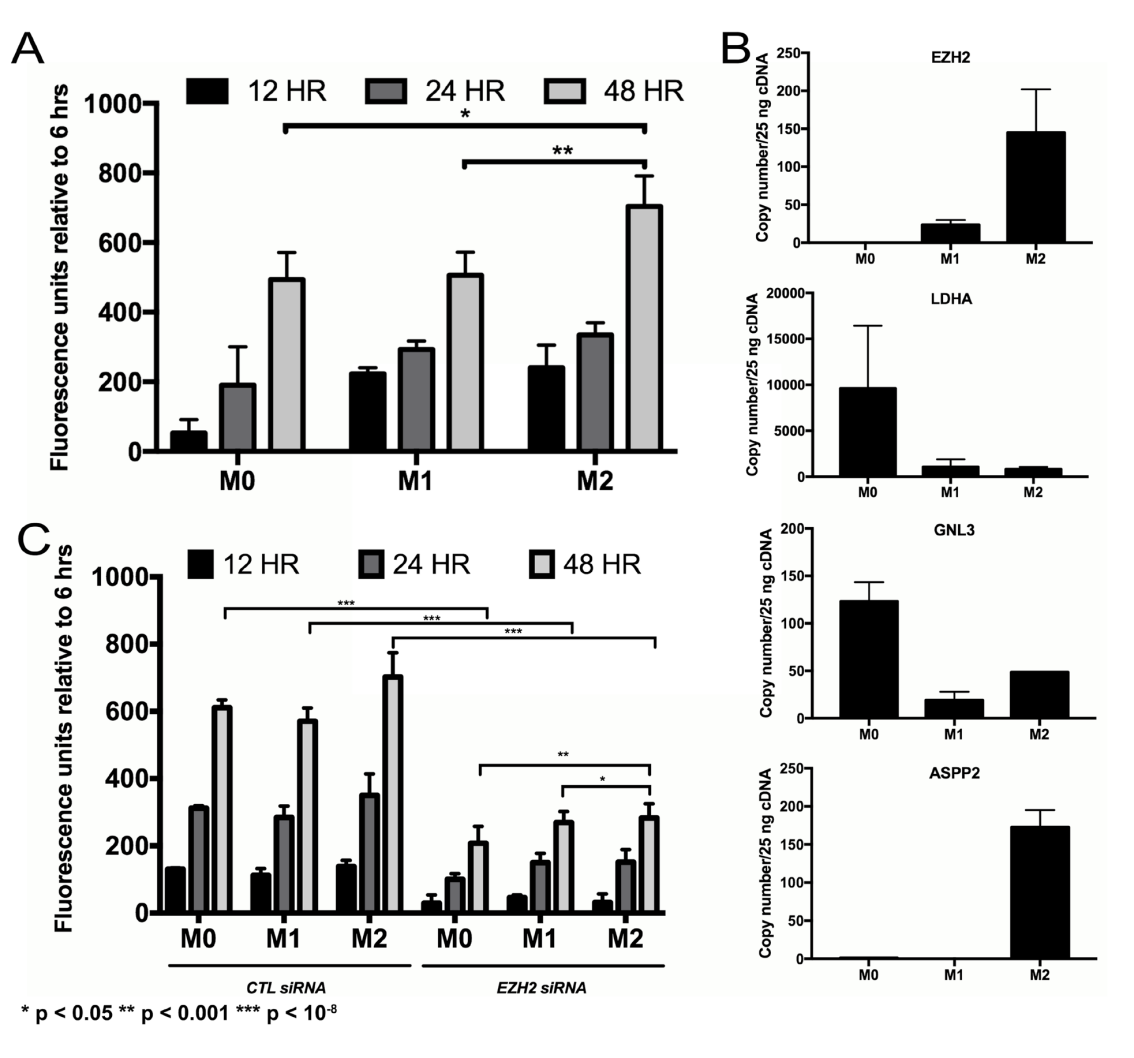
Impact of TAMs on NFPA cell proliferation (**A**) Conditioned media from THP1 human monocytes differentiated into macrophages (unpolarized, M0; polarized, M1 and M2) was applied to primary NFPA cells and it was found that M2 polarized macrophages promoted greater proliferation of these cultures over time than M1 macrophages (Student's *t*-test, *P* < 0.001). (**B**) Quantitative PCR assessment of potential proliferation-mediating genes in NFPAs revealed that *EZH2* was the only target in which conditioned media from M2 macrophages increased gene expression over M1 CM, matching the trend seen in proliferating cells. (**C**) siRNA targeting of *EZH2* eliminated most of the proliferative increases seen in NFPA cultures over time (Student's *t*-test, *P* < 0.05).

CM from THP-1 human monocytes treated and polarized to M2 macrophages promoted greater proliferation of primary NFPA cultures than CM from M1-polarized macrophages (*P* < 0.001; Figure 3A). Follow-up qPCR assessment of potential proliferation-mediating genes in NFPAs revealed that only *EZH2* demonstrated increased expression in NFPAs grown in CM from M2 macrophages as compared to NFPAs grown in CM from M1 macrophages (Figure 3B). Targeted knockdown of *EZH2* expression via siRNA gene silencing significantly reduced proliferation of cultured primary NFPA cells, including reducing the proliferation increase seen in cells with M2 CM (*P* < 0.05; Figure 3C).

### Effects of TAMs on NFPA invasion and motility

**Figure 4:**
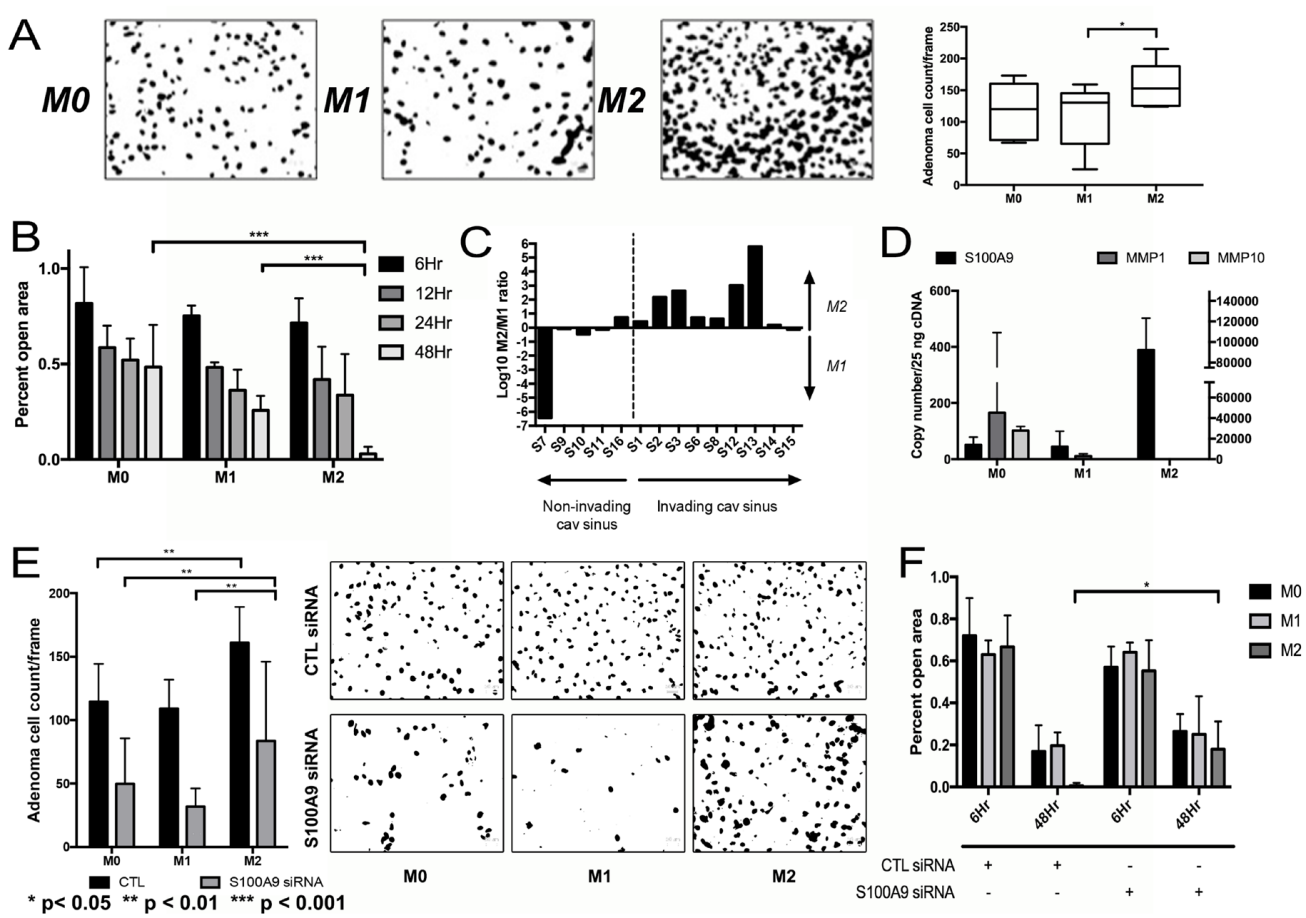
Impact of TAMs on NFPA cell migration (**A**) CM from M2 macrophages developed from THP-1 human monocytes promoted greater invasion as seen in matrigel chamber assays than CM from M1 macrophages (*P* < 0.05). (**B**) Motility assessed using cell scratch images taken in quadruplicate found that CM from M2 macrophages promoted greater motility than CM from M1 macrophages (*P* < 0.001). (**C**) qPCR for three M1 and three M2 markers performed on FACS-isolated CD11b+ TAMs from NFPA patient specimens to derive an M2/M1 ratio revealed that 8 of 9 tumors with cavernous sinus invasion had M2/M1 gene expression ratios above one (log (M2/M1)>0), while just 1 of 5 tumors without cavernous sinus invasion had M2/M1 ratio above one (*P* = 0.02). (**D**) qPCR screening was performed on cells from a primary NFPA patient using potential markers previously described to mediate invasion or motility in other cancer types. Of the markers screened, *S100A9* demonstrated a notable increase in gene expression in cells growing in M2. (**E**, **F**) siRNA targeting of *S100A9* inhibited both the invasion (*P* < 0.01) and motility (*P* < 0.05) effects of macrophage CM on primary NFPA cultures. All *p*-values calculated using Student's *t*-test.

We next examined the effects of macrophage CM on pituitary cell invasion and motility. CM from polarized M2 macrophages developed from THP-1 human monocytes promoted greater invasion in matrigel chamber assays (*P* < 0.05; Figure 4A) and motility in scratch assays (*P* < 0.001; Figure 4B) of primary NFPA cultures than CM from M1 macrophages derived from THP-1 cells. Consistent with this finding, we performed qPCR for the three M1 and three M2 markers listed above on FACS-isolated CD11b^+^ TAMs from our NFPA patient specimens to derive an M2/M1 ratio as we have described previously [23] and revealed that 8 of 9 tumors with cavernous sinus invasion had M2/M1 gene expression ratios above one (log (M2/M1)>0), while just 1 of 5 tumors without cavernous sinus invasion had M2/M1 ratio above one (*P* = 0.02; Table 1; Figure 4C). Assessment of potential invasion-mediating genes in NFPAs revealed that CM from M2 macrophages displayed increased expression of *S100A9*, a regulator of inflammation and invasion expressed by cancer cells (Figure 4D). Targeting of *S100A9* via siRNA as described above inhibited the invasion (*P* < 0.01; Figure 4E) and motility (*P* < 0.05; Figure 4F; Supplementary Figure 3) of primary NFPA cultures under all conditions, including in M2 CM.

### Effects of NFPAs on macrophages

**Figure 5:**
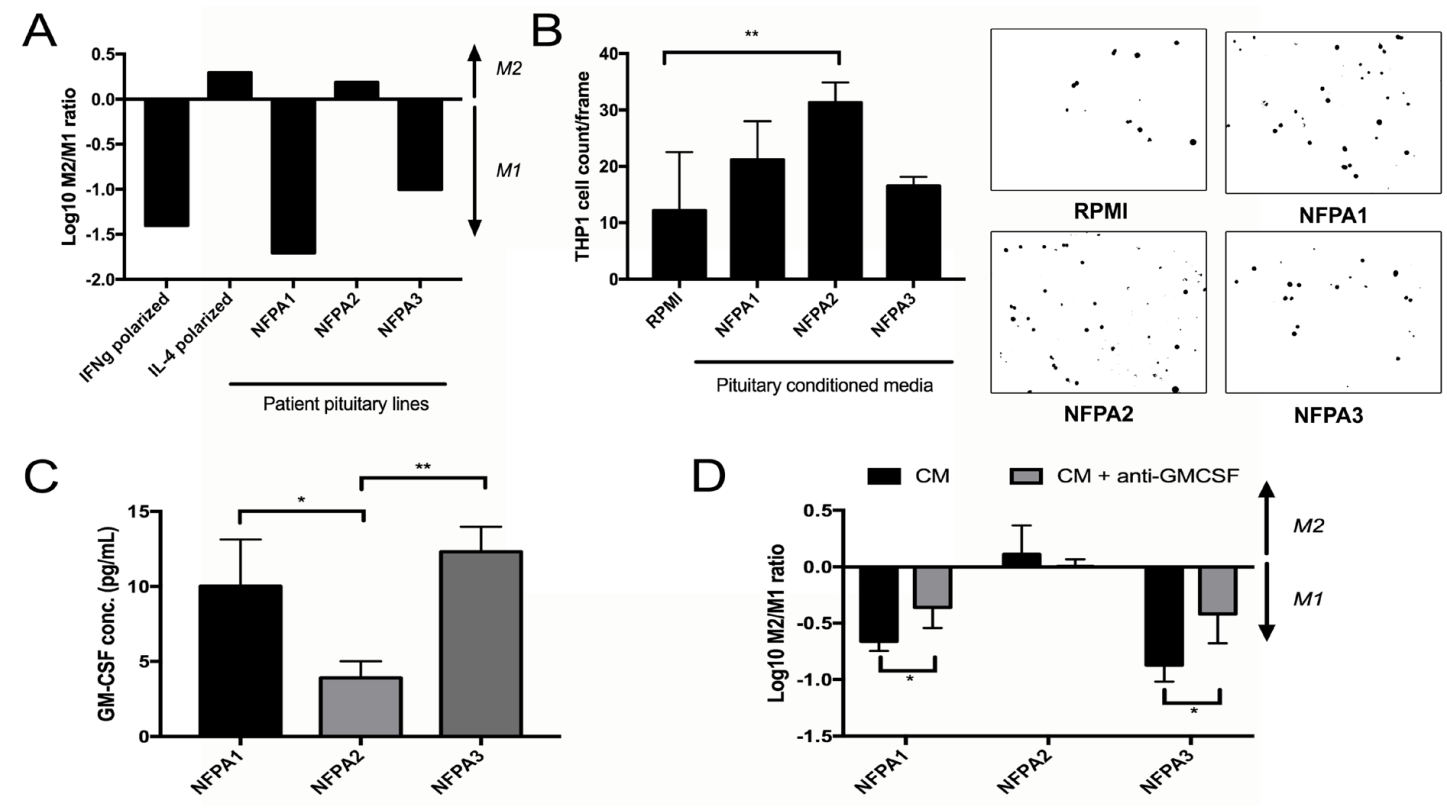
Impact of NFPA cells on TAMs (**A**) Primary NFPA cultures (*n* = 3) were utilized to obtain CM, which was subsequently applied to THP-1 cells to measure polarization effects by the log of the M2/M1 gene expression ratio. Two of the analyzed NFPA cultures promoted M1 polarization of THP-1 cells, while the third promoted M2 polarization, with IFNγ and IL-4 applied to THP-1 cells as positive controls for M1 and M2 polarization, respectively. (**B**) Invasion assessed by matrigel chamber assay found that the NFPA culture that promoted M2 polarization (NFPA2) attracted more THP-1 monocytes than the two NFPA cultures that promoted M1 polarization (*P* < 0.001). (**C**) A multiplex ELISA analyzing levels of eight chemokines related to M1/M2 polarization was performed on media from our three primary NFPA cultures. Results are in Supplementary Figure 2. Results for GM-CSF are shown here, and revealed that GM-CSF levels were uniquely elevated in media from NFPA1 and NFPA3 cells compared to NFPA2 cells (*P* < 0.05 and *P* < 0.001, respectively). (**D**) Blocking of this protein utilizing a GM-CSF neutralizing antibody in NFPA CM reversed much of the M1 polarization caused by NFPA1 and NFPA3 cells (*P* < 0.05). All *p*-values calculated using Student's *t*-test.

Primary NFPA cultures (*n* = 3) were then used to generate pituitary CM, which was subsequently applied to THP-1 cells in the resting M0 macrophage stage to determine the effect of NFPA-secreted factors on macrophage polarization. Two of the analyzed NFPA cultures (NFPA1 and NFPA3) promoted M1 polarization of THP-1 cells, while the third analyzed NFPA culture (NFPA2) promoted M2 polarization of THP-1 cells (Figure 5A). We then analyzed the chemotactic attraction of THP-1 monocytes to these NFPA cultures and found that the NFPA culture that promoted M2 polarization attracted more monocytes than the two NFPA cultures that promoted M1 polarization (*P* < 0.001; Figure 5B). To identify potential mediators of the M1 polarization and reduced monocyte chemotaxis that we found to be promoted by NFPA1 and NFPA3 but not by NFPA2, we analyzed levels of a panel of macrophage-relevant chemokines in the media of these NFPA cultures using a commercially available chemokine multiplex ELISA kit and found GM-CSF protein levels to be uniquely elevated in media from NFPA1 and NFPA3 cells compared to NFPA2 cells (*P* < 0.05 and *P* < 0.001, respectively; Figure 5C; Supplementary Figure 4). To investigate the role of GM-CSF in NFPA-induced macrophage polarization, a GM-CSF neutralization antibody was applied to primary NPFA cultures before CM collection. The resultant CM was applied to THP-1 cells before cell harvesting for RNA and qPCR analysis of our previously described M1 and M2 markers. The results of this qPCR showed that NFPA1 and NFPA3 lost significant levels of their M1 polarization as characterized by gene expression (*P* < 0.05) (Figure 5D). These findings suggested that GM-CSF was driving reduced monocyte chemotaxis and M1 polarization in a subset of NFPAs. Unlike MCP-1, these changes in GM-CSF did not carry over into the serum from these patients, making its utility as a potential NFPA immune biomarker less than that of MCP-1 (Supplementary Figure 5).

## DISCUSSION

**Figure 6:**
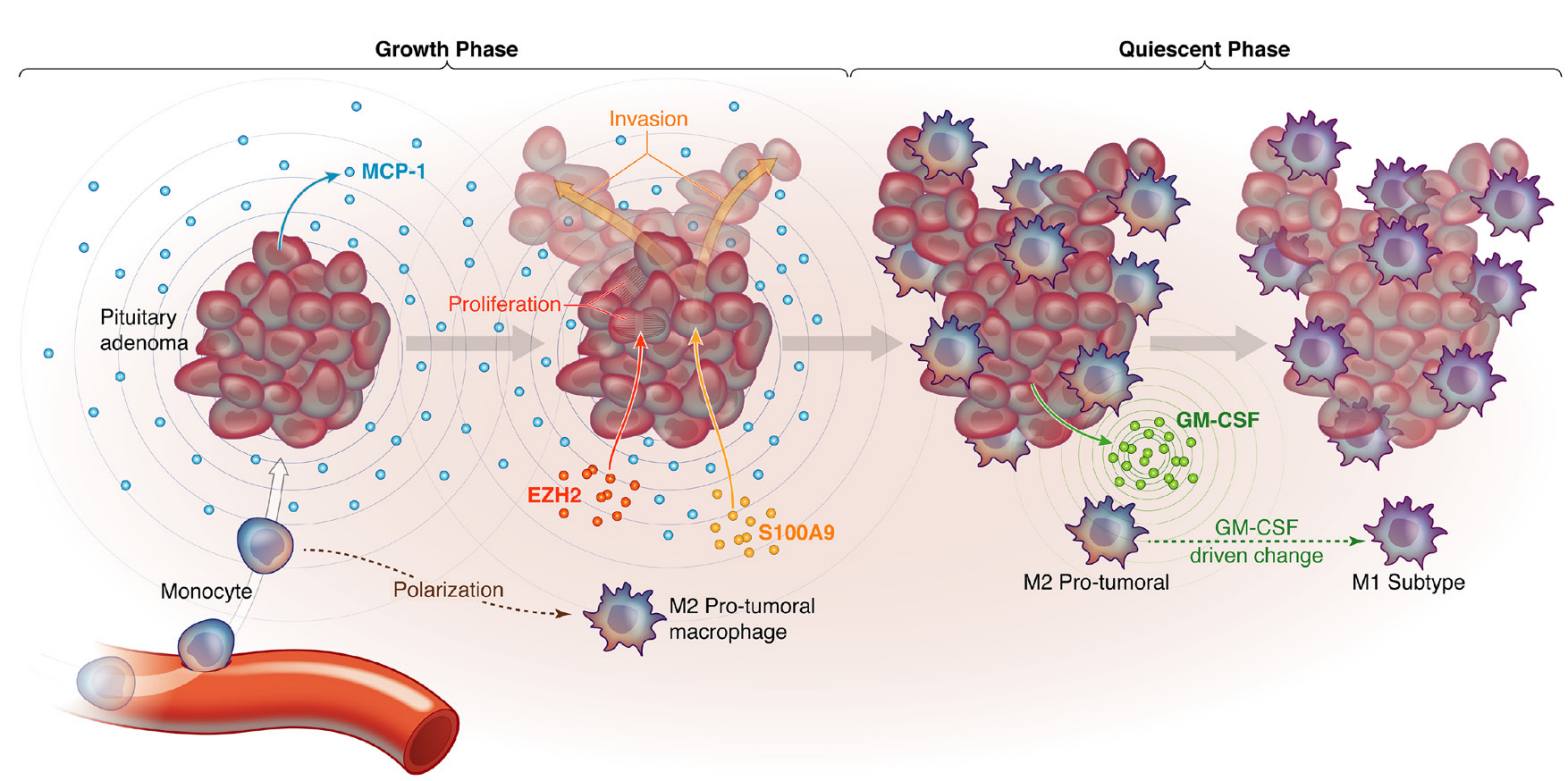
Proposed model for how macrophages are recruited to NFPAs in a manner that creates distinct growth phases Shown are a proposed NFPA growth phase during which NFPA cells secrete MCP-1 to attract monocytes to the adenoma, which are then differentiated into macrophages and polarized to pro-tumoral M2 macrophages, which in turn drive NFPA cells to express EZH2, driving proliferation, and S100A9, driving invasion. This is followed by a proposed NFPA quiescent phase, during which NFPA cells transition to GM-CSF secretion, converting M2 TAMs to M1 TAMs that curb tumor growth.

Our study is the first to report an association between TAM subtypes and NFPA behavior in culture and in patient samples. We found two distinct subtypes of NFPAs based on TAM status. The first type attracted more monocytes due to more MCP-1 expression and polarized the recruited macrophages towards an M2 subtype that promotes tumor cell proliferation and invasion, while the second type attracted fewer monocytes due to less MCP-1 expression and polarized the resulting macrophages towards an M1 subtype due to more GM-CSF expression. While NFPAs cannot be sampled serially over time, we hypothesize based on our data that NFPAs start out as the first type, attracting monocytes that they convert to TAMs and M2 polarize, enabling tumor proliferation and invasion, but, over time, NFPAs transition to the latter type as the M2 TAMs suppress NFPA MCP-1 production so that the NFPAs slow their monocyte attraction and the NFPA cells increase their GM-CSF production driving the TAMs that are present to an M1 subtype which is less supportive of tumor growth and invasion (Figure 6). This feedback loop ensures that most NFPA growth occurs early on but the microenvironment eventually reigns in this growth. Such a hypothesis would be consistent with the logistic mathematical model of tumor growth: an exponential phase followed by a linear phase and lastly, a plateau phase. The best radiographic demonstration of such a growth pattern to date for NFPAs was a study in which 5 of 15 cases with initial exponential growth showed decelerated growth during the observation period consistent with a logistic growth pattern [24].

Because the fenestrated capillary network present within the hypothalamic-hypophyseal portal circulation position the pituitary gland outside the blood-brain barrier, the pituitary gland has never been regarded as immunoprivileged the way that the rest of the central nervous system is. It is thus not surprising that an increasing number of studies have demonstrated a rich immune infiltrate surrounding both functional and nonfunctional tumors. Macrophages, T, and B cells appear to be present in differing fractions within pituitary adenoma. Our study advances our understanding of the role of these cells in NFPA pathogenesis.

Our TAM subtype analysis underscored some themes that have been reported in other cancers. When relying on three M1 and three M2 markers via qPCR of the CD11b fraction, we were able to correlate the M1-M2 TAM distribution with clinical NFPA behavior, underscoring the importance of analyzing these fractions via multiple antigens because the M1-M2 distribution may be a continuous spectrum rather than dichotomous extremes [13]. Our finding that M1 and M2 fractions analyzed by flow cytometry did not sum to the entire CD11b fraction is also likely due to this spectrum as well as the presence of M0 TAMs that have not yet polarized into a specific subtype [22].

We identified EZH2 as a mediator of M2-induced NFPA proliferation. EZH2 is an epigenetic regulator whose role in cell cycle regulation stems from it being a principal component of polycomb repressive complex 2, a protein complex known to methylate lysine residue 27 of histone 3 (H3K27), enabling it to prevent cellular senescence and prolonging cell survival in a variety of cancers) [25–28]. A small but growing literature has shown that EZH2 is expressed in pituitary adenoma cells in a manner that correlates with Ki-67 labeling but not in the normal pituitary gland [29], with likely roles in adenoma proliferation and possibly angiogenesis [30].

Similarly, our work identified S100A9 as a mediator of M2 TAM-induced invasion. S100A9 is a member of the S100 family of calcium-binding proteins that may serve as an inhibitor of casein kinase. S100 proteins have been shown to be expressed in pituitary adenomas in a manner that correlates with VEGF and EGFR expression [31]. Furthermore, while S100 proteins have not been directly linked to invasion in pituitary adenomas, the S100A8 and S100A9 proteins have been shown to mediate TAM-induced metastases in colon and lung cancer cell lines [32] and their heterodimer protein, calprotectin, has been found to induce members of the NF-kB and p38 pathways in macrophages, stimulating increased release of pro-inflammatory cytokines [33].

Our work thus suggests that EZH2 and S100A9 may warrant investigation as therapeutic targets in growing NFPAs that are not curable with surgery. In addition, our work could also have translational benefits if it gave rise to immunomodulatory therapeutic approaches for these large NFPAs. These approaches could include therapies promoting M1 macrophage polarization such as antibodies targeting colony-stimulating factor 1 receptor (CSF1R), which are undergoing clinical trials [34].

## MATERIALS AND METHODS

### Patient sample collection

Fresh tissue from non-functional pituitary adenomas were obtained from surgeries performed at University of California San Francisco under an IRB-approved protocol (11-05901). The Brain Tumor Research Center assisted in sample collection and obtaining of patient consent under their parental IRB-approved protocol (10-01318). Tissue samples were retained in DMEM + 10% FBS at explantation. This fresh tissue was assigned a unique numerical identifier (S1-16 for samples undergoing flow cytometry, SDB1-3 for samples undergoing flow cytometry of site-directed biopsies, and NFPA1-3 for samples put in culture) for PHI protection. In parallel, UCSF neuropathologists confirmed the diagnosis of pituitary adenoma and performed MIB1-labeling (Dako, M7240, 1:100) of paraffin-embedded sections from these cases as is our standard institutional practice for resected adenomas.

### Flow cytometry

A tissue dissociation protocol was carried out on fresh tissue obtained from surgical cases. Tissue was manually minced with a razor blade and incubated in papain dissociative agent (Worthington Biochemical; LS003118) for 30 minutes. The supernatant was then passed through a 40 micron filter to obtain a single cell suspension. Cells were subsequently treated with a red blood cell (RBC) lysis buffer (Thermo Fisher; #00-4333-57) and the remaining cells were counted and resuspended in FACs buffer (HBSS + 2% sterile FBS). Antibodies were added at the appropriate concentration following the information listed in Supplementary Table 1 and incubated for 30 minutes before washing twice with FACs buffer and resuspending for flow cytometry analysis. Flow cytometers were reserved via the UCSF Laboratory for Cell Analysis core and samples were processed on a BD FACSAria 3 or a Sony SH800, depending on availability. Compensation was performed using comp beads (Invitrogen OneComp eBeads; 01-1111-42) on both machines and similar gating practices were followed to determine macrophage percentage, as well as M1 and M2 fractions.

### Cell culture

THP-1 human monocytes (ATCC TIB-202) were passaged fewer than six months, verified by the providing company using short tandem repeat (STR) profiling and confirmed to be mycoplasma free. Cells were cultured in RPMI supplemented with 10% FBS and 1% of the following: HEPES buffer, sodium pyruvate, PenStrep antibiotic, non-essential amino acids (NEAA) and GlutaMax glucose solution. To establish primary pituitary adenoma cells in culture, an NFPA was provided fresh from the operating room by the UCSF Neurosurgery Tissue Bank. The tumor was mechanically dissociated with a razor blade in 150 μL digestion buffer. Enzymatic digestion buffer consisted of RPMI-1640 (UCSF Cell Culture Facility) with 10% FBS (UCSF Cell Culture Facility; San Francisco, CA, USA), 100 μL/mL DNase I (Roche Diagnostics; Indianapolis, IN; #10104159001), and 200 U/mL Collagenase IV (Life Technologies; San Francisco, CA, USA),; #17104019). Once fine, tissue suspensions were transferred to a vial containing 7 mL digestion buffer and incubated at 37° C for 45 min. while rotating. Tumor suspensions were then washed with 2% FBS in DPBS (UCSF Cell Culture Facilities) and passed through a 70 μm cell strainer (Corning; Corning, NY; #352350). Unstrained tumor chunks were transferred to a 100 mm petri dish (Corning; #430167) with DMEM + 10% FBS + 100 U/mL Penicillin and 100 μg/mL Streptomycin (UCSF CCF). Strained cells were washed and pelleted with 2% FBS in DPBS twice before treatment with 1x RBC Lysis Buffer by manufacturer's instructions (eBioscience; San Diego, CA, USA),; #00-4300-54). Cell suspensions were then washed with 5% FBS in DPBS, pelleted, and plated on T-25 flasks at 3.5 × 10^5^ viable cells/mL in complete DMEM.

### Conditioned media

#### THP1 monocytes

Undifferentiated monocytes were seeded at 2 × 10^6^ cells/well in a 6-well dish in RPMI media supplemented with 10% FBS, 0.05% 2-ME and 1% of each of the following: HEPES buffer, PenStrep solution, NEAA, GlutaMax and sodium pyruvate. PMA (phorbol myristate acetate) was added at 50 ng/uL to allow THP1 cell adhesion to the plate. Cells were incubated at 37° C for 4 days to become resting M0 macrophages. At this stage, M0 CM could be collected or polarization supplements were added. Polarization to M1 or M2 macrophages was achieved with the addition of 50 ng/ul of purified IFNγ or IL-4, respectively. Cells were incubated for 24 additional hours with polarization agent before collection of CM. All conditioned media was then centrifuged at 300 × *g* for 5 minutes and the supernatant was drawn off and saved at −20° C.

#### Primary pituitary cells

Cells were seeded at subconfluent levels (~50% confluency) and incubated at 37° C for 2 days in DMEM + 10% FBS and 1% PenStrep. After two days, conditioned media was removed from cells and spun down as described above before storage at −20° C.

### qPCR

RNA isolated with an RNeasy kit (Qiagen) was reverse-transcribed into cDNA with qScript XLT cDNA SuperMix (QuantaBio; #95161-025). Powerup Syber Green Master Mix (Thermo Fisher; #A25741) was used with primers described in Supplementary Table 2. Absolute quantitative real-time PCR was performed by generating a standard curve was constructed by plotting Ct values against logarithmic concentrations of a serially diluted *ACTB* plasmid, dilutions ranging between 10^6^ and 10^1^ copies per reaction. Samples of interest were prepared as described for the relative quantification, with *ACTB* qPCR primers used as a control. Results were adjusted for qPCR efficiency and exact copy numbers of unknown samples were quantified by interpolating Ct values against the standard curve. All reactions were carried out on a StepOne RealTime PCR machine and Ct values generated for analysis using the corresponding StepOne software. M1 value was determined by calculating the averaged expression level of three separate M1 markers (Nos2 = inducible nitric oxide synthase, CXCL10, and IL-1β), while M2-specific markers (Arginase 1, TGF-β, and MMP9) were similarly used to calculate the M2 value. M2/M1 ratios were calculated using these values.

### siRNA

Targeted knockdown via siRNA was carried out using commercially available siRNA pools for genes of interest (*S100A9*, Santa Cruz Biotechnology; #sc-43344; *EZH2*, ThermoFisher; #16708). The siRNA was resuspended according to manufacturer's guidelines. For transfection purposes, the FuGENE6 transfection reagent (Promega; #E2691) protocol was used to calculate the amount of reagent needed for 1 μg siRNA (for each well of a scratch assay) or 0.2 μg siRNA (for each Boyden chamber used for the matrigel invasion assays). The volume of the mix was brought up to 100 μL with serum-free media. The transfection mix was allowed to incubate for 30 minutes before spiking into primary NFPA cells (2 × 10^6^ for scratch assay, 5 × 10^5^ for invasion assay). Cells and siRNA mixture were then seeded into appropriate vessel (6 well plate for scratch assay, upper level Boyden chamber for invasion assay) and conditioned media could be added when appropriate.

### Transwell matrigel invasion assay

Experiments to compare invasion rates of NFPA cells and chemotactic attraction of THP-1 cells to NFPA cells were conducted using a Boyden chamber transwell system (8 μm PET membrane, Becton Dickinson; San Diego, CA, USA; #354578), which separated plates into upper and lower chambers. Matrigel was thawed on ice was added quickly with cold tips to cold PBS at a 1:25 dilution, pipetted onto the base of the chamber inserts, dried, and inverted back into the 24 well plates. NFPA or THP-1 cells were seeded at a density of 5 × 10^5^ cells/well in upper chambers in the appropriate media. NFPA media was placed in the lower chamber for NFPA invasion assays, and NFPA cells in their media were placed in the lower chamber for THP-1 chemotaxis assays. At migration time points, inserts were removed and washed of media before drying for 2 hours. The matrigel membrane was cut off the insert and placed on a slide for imaging with DAPI reagent. Imaging was carried out using a Zeiss Axio Observer inverted microscope (Zeiss; Gottingen, Germany) outfitted with an Axi.Observer.Z1m stand and AxioVision software. Cells in each 40x frame were counted using ImageJ software (Version 1.5, National Institutes of Health; Bethesda, MD) [35]. Files were first converted to 16-bit grayscale images, then the threshold was manually adjusted to highlight cells while reducing background noise. The resultant image was then examined and noticeably overlapping cells were separated using a white line divider. Finally, cells were counted using ImageJ's automated “Analyze Particles” feature.

### Scratch assay

Pituitary adenoma cells were seeded at 75% confluency on the wells of a 6 well plate and allowed to grow to ~90% confluency. At time = 0, media was aspirated off, cells were washed with PBS and conditioned media was replaced according to the required conditions (M0, M1, M2). A P200 pipette tip was used to create a wound in the cell layer from top to bottom. Positional definition used to take photos in the same place each time was achieved by marking the underside of the plate with two lines perpendicular to the direction of the wound. Images were taken at the wound site above and below each drawn line (for a total of four replicates/condition/time point). Once imaged, cells were returned to 37° C incubation until next time point occurred. Percent of open area in each image was analyzed using the ImageJ macro “Wound Healing Tool” (http://dev.mri.cnrs.fr/projects/imagej-macros/wiki/Wound_Healing_Tool) and values were normalized to open area as seen at time = 0.

### CyQuant assay

Pituitary adenoma cells were plated at a density of 5 × 10^4^ cells/well in a 96 well culture plate, then incubated at 37° C for multiple independent time points. At the specified time point, plate was removed from 37° C incubation, centrifuged for 3 minutes at 3000 rpm and each well was aspirated of media. Plates were then frozen at −80° C for at least 2 hours (longer times permitted). Once all time points were frozen, plates were brought to room temperature and proliferation assays were carried out using the CyQUANT Cell Proliferation Assay Kit (ThermoFisher #C7026), following standard manufacturer's protocol.

### ELISA

ELISA assays utilized for this experiment were premade with attached antibodies for MCP-1 (ThermoFisher; #BMS281), GM-CSF (ThermoFisher; #BMS283), or a panel of seven previously defined cytokines related to M1 an M2 macrophage polarization (IFNγ, IL-4, IL-6, IL-10, IL-12, MCAF, and TNF-α) (Anogen; #EM10002). ELISA samples were either banked serum collected during patient surgery or CM from three primary NFPA lines collected as described above. Stock solution containing known concentrations of all cytokines was provided with ELISA kits and diluted according to manufacturer's recommendations to create standards for quantitation. Standards and samples were added in triplicate to appropriate wells and incubated for one hour, then bound with biotin conjugate and washed following manufacturer's protocol. Plate was read in a microtiter plate reader set to OD 450 nm.

### Statistics

All experiments were performed with three biologic and technical replicates. All statistical analysis was performed using SPSS (IBM SPSS Statistics Developer 25.0) with significance defined as *P* < 0.05. Chi-square and Fisher's exact test were used for categorical variables, the student's *T* test for continuous variables.

## SUPPLEMENTARY MATERIALS FIGURES AND TABLES



## Abbreviations

CD: Cluster of differentiation
EZH2: Enhancer of zeste homologue 2
NFPA: Non-functional pituitary adenoma
siRNA: small interference ribonucleic acid
TAM: Tumor-associated macrophage
