# Correction: Stratifying nonfunctional pituitary adenomas into two groups distinguished by macrophage subtypes

**DOI:** 10.18632/oncotarget.27048

**Published:** 2019-07-02

**Authors:** Garima Yagnik, Martin Rutkowski, Sumedh S. Shah, Manish K. Aghi

**Affiliations:** ^1^ Department of Neurosurgery, University of California San Francisco (UCSF), San Francisco, CA, USA

**This article has been corrected:** The proper spelling of the 2nd author’s name is ‘Martin Rutkowski’.

**Martin Rutkowski**

Original article: Oncotarget. 2019; 10:2212–2223 . https://doi.org/10.18632/oncotarget.26775

