# Supplementary material for: Stratifying nonfunctional pituitary adenomas into two groups distinguished by macrophage subtypes
Source: Oncotarget. 2019 Mar 15;10(22):2212–23. doi: 10.18632/oncotarget.26775 (PMC6481336; doi:10.18632/oncotarget.26775)
Supplement: Supplementary file 1 [file oncotarget-10-2212-s001.pdf]

Stratifying nonfunctional pituitary adenomas into two groups distinguished by macrophage subtypes

SUPPLEMENTARY MATERIALS

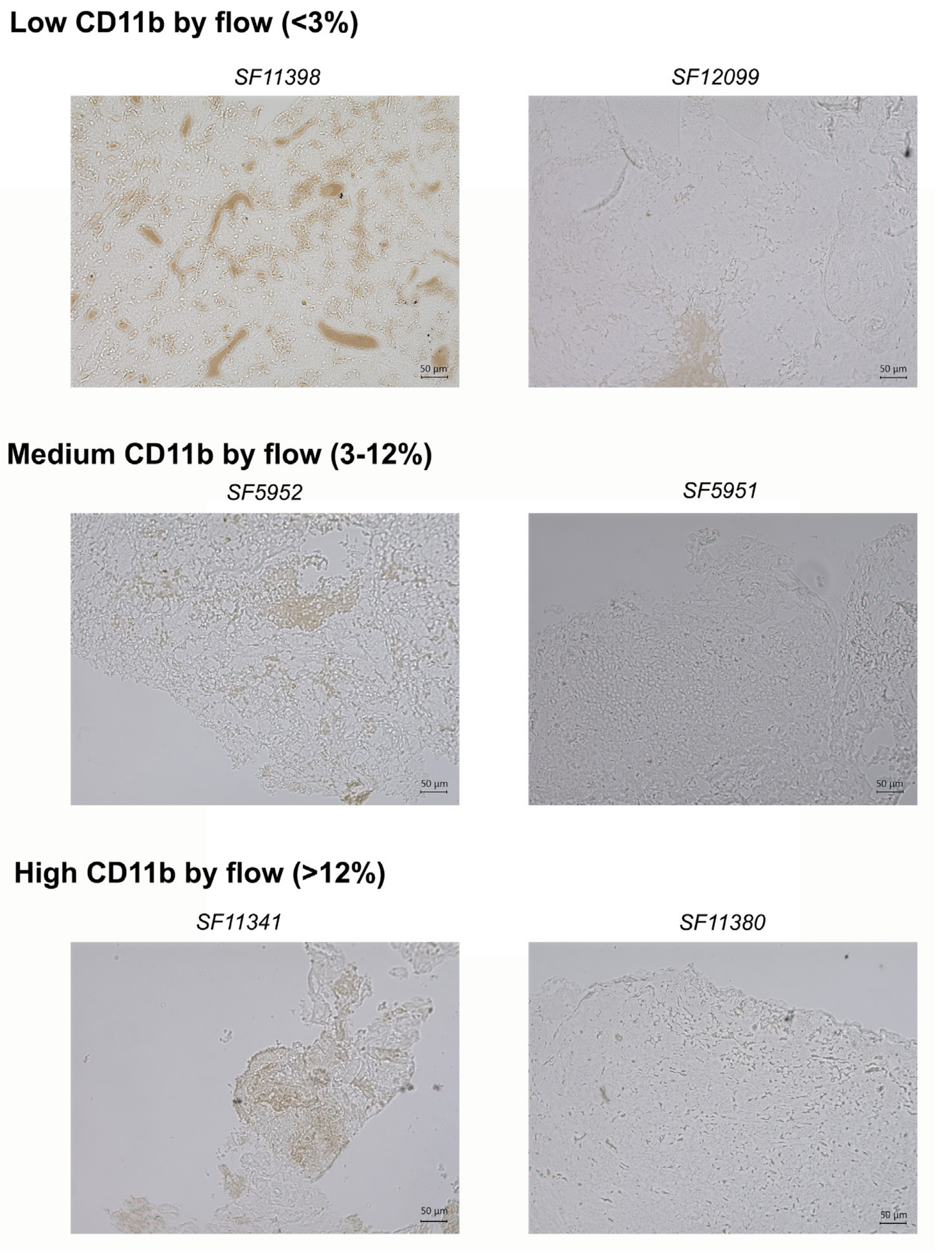

Supplementary Figure 1: Immunohistochemistry staining for CD11b. Related to Figure 1B. Tissue sections from NFPA cases deemed to have high, medium or low CD11b percentage by flow cytometry were stained for CD11b with an immunohistochemistry (IHC)-compatible antibody. Results did not correlate well with those seen with flow cytometry. Representative images from two cases in each group are presented. Scale bars, 50 mm.

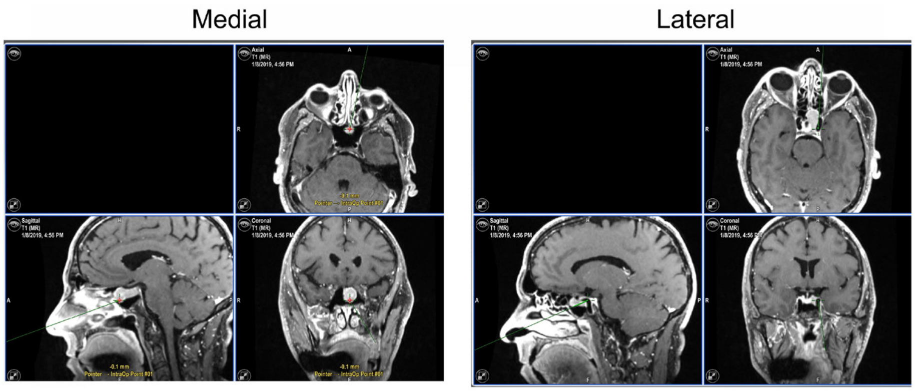

Supplementary Figure 2: Representative screenshots from site-directed biopsies of a nonfunctional pituitary adenoma. Related to Figures 1C and 2C. Shown are neuro-navigation screenshots illustrating representative paired site-directed biopsies from a single patient with a nonfunctional pituitary adenoma. A medial sample was taken from the anterior central portion of the adenoma, and a lateral sample was taken from the lateral (in this example, left) portion of the adenoma abutting the medial cavernous sinus wall. This protocol was followed to obtain site-directed biopsies from four nonfunctional adenomas in this study.

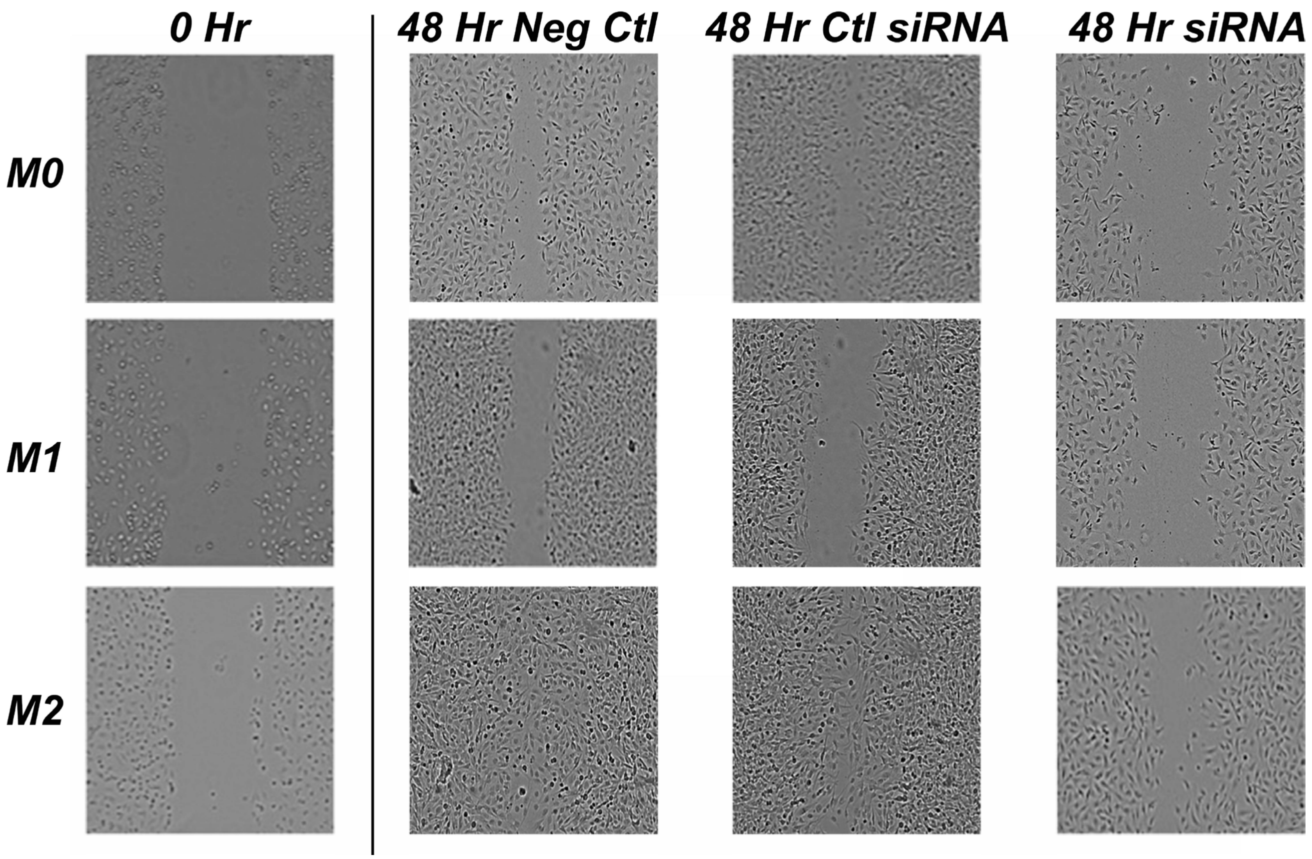

Supplementary Figure 3: Scratch assay images from NFPA cells grown in macrophage conditioned media. Related to Figure 4F. Shown are raw images from the scratch assays performed on NFPA cells exposed to M0, M1, or M2 CM after expressing no siRNA (neg Ctl), siRNA targeting control sequences (Ctl siRNA), or siRNA targeting S100A9 (siRNA).

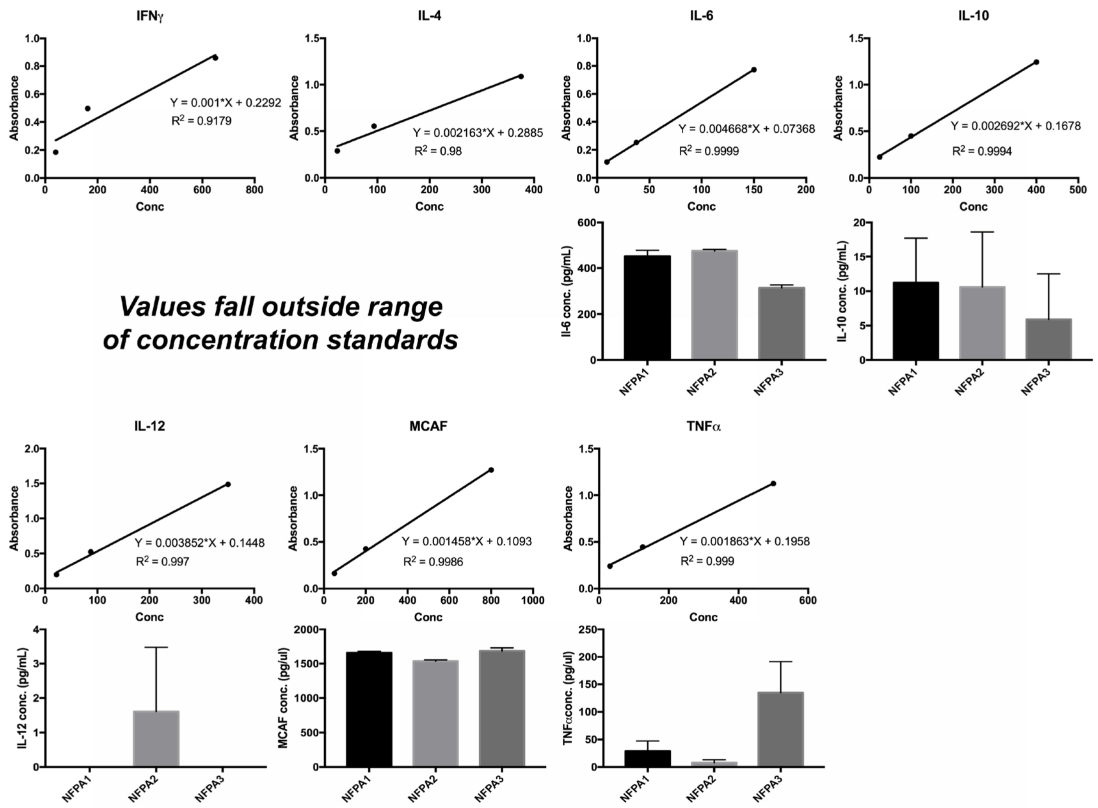

Supplementary Figure 4: Multiplex chemokine ELISA results from NFPA cell media. Related to Figure 5C. Shown are results of ELISAs performed using a multiplex chemokine array to detect NFPA produced IFNγ, IL-4, IL-6, IL-10, IL-12, MCAF, and TNF-α.

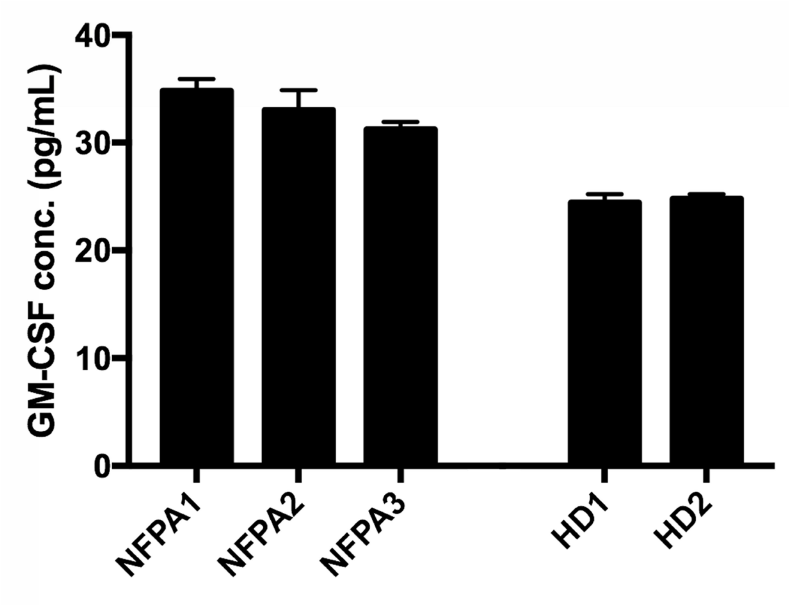

Supplementary Figure 5: Human GM-CSF ELISA results from NFPA cell line paired patient serum. Related to Figure 5C. Serum collected at time of tumor explantation from patients whose NFPAs were used to establish three cell lines underwent GM-CSF ELISAs. Results revealed minimal elevation of serum GM-CSF in NFPA patients compared to two healthy donors (HD) and no trend resembling GM-CSF levels of the cultured cells.

Supplementary Table 1: Antibodies used for flow cytometry

Cell marker target	CD11b	CD206	CD64
Clone	ICRF44	15-2	10.1
Fluorochrome	AlexaFluor 488	Brilliant Violet 421	APC
Vendor	BioLegend	BioLegend	BioLegend
Catalog Number	301318	321126	305014
Lot Number	B223756	B240241	B229885
Dilution	5 uL/10^6 cells	5 uL/10^6 cells	5 uL/10^6 cells

Detailed information on antibodies used for flow cytometry experiments

Supplementary Table 2: Primers used for qPCR

Gene target	Forward	Reverse
CCL2	5′ – CAG CCA GAT GCA ATC AAT GC – 3′	5′ – GCA CTG AGA TCT TCC TAT TGG TGA A – 3′
EZH2	5′ – TTT CCA GAT AAG GGC ACA GC – 3′	5′ – AAG TGT TGG GTG TTG CAT GA – 3′
LDHA	5′ – GGT TGG TGC TGT TGG CAT GG – 3′	5′ – TGC CCC AGC CGT GAT AAT GA – 3′
GNL3	5′ – GAA GCC TCC GAT GTT GTC CT – 3′	5′ – CAC ACG CTTG GTT ATC TTC CC – 3′
ASPP2	5′ – ATC ACG GCT CTT CAC AAT GC – 3′	5′ – ATG TCA CTG TAG GTC ATG GCA – 3′
S100A9	5′ – CCA TCA TCA ACA CCT TCC ACC – 3′	5′ – TGA TGA ACT CCT CGA AGC TCA – 3′
MMP1	5′ – CTA CAC GGA TAC CCC AAG GAC – 3′	5′ – CAT CAA CTT TGT GGC CAA TTC – 3′
MMP10	5′ – GGT TAT CCA AGA GGC ATC CAT – 3′	5′ – AGG CTC AAC TCC TGG AAA GTC – 3′
ARG1	5′ – CCC TTT GCT GAC ATC CCT AA – 3′	5′ – GAC TCC AAG ATC AGG GTG GA – 3′
TGFB1	5′ – CAC GTG GAG CTG TAC CAG AA – 3′	5′ – TGC AGT GTG TTA TCC CTG CT – 3′
MMP9	5′ – TTG ACA GCG ACA AGA AGT GG – 3′	5′ – GCC ATT CAC GTC GTC CTT AT – 3′
NOS2	5′ – AGC ATG TAC CCT CGG TTC TG – 3′	5′ – GGG GAT CTG AAT GTG CTG TT – 3′
CXCL10	5′ – TGG CAT TCA AGG AGT ACC TCT C – 3′	5′ – TTC TTG ATG GCC TTC GAT TC – 3′
IL1B	5′ – ACG GAT TCC ATG GTG AAG TC – 3′	5′ – GAG TGT GGA TCC CAA GCA AT – 3′
GAPDH	5′ – GCT GAG AAC GGG AAG CTT GT – 3′	5′ – TCT CCA TGG TGG TGA AGA CG – 3′
ACTB	5′ – ACT CTT CCA GCC TTC CTT CC – 3′	5′ – TGA TCT TCA TTG TGC TGG GTG – 3′

Shown are primers used for qPCR of human genes.
